# Excess soluble alkalis to prepare highly efficient MgO with relative low surface oxygen content applied in DMC synthesis

**DOI:** 10.1038/s41598-021-00323-5

**Published:** 2021-10-22

**Authors:** Ju Liu, Fei Chen, Wenbing Yang, Jianjun Guo, Guangwen Xu, Fenglei Jia, Lei Shi

**Affiliations:** 1 Key Laboratory On Resources Chemicals and Materials of Ministry of Education, Shenyang University of Chemical Technology, Shenyang, 110142 China; 2 Shandong Shida Shenghua Chemical Group, Dongying, 257000 China; 3 Institute of Industrial Chemistry and Energy Technology, Shenyang University of Chemical Technology, Shenyang, 110142 China

**Keywords:** Catalysis, Chemical synthesis

## Abstract

The activities of various MgO catalysts, which were prepared from different methods such as hydration synthesis, thermal decomposition, combustion, sol–gel and co-precipitation, were conducted in dimethyl carbonate (DMC) synthesis via transesterification of ethylene carbonate with methanol. MgO-P-Na_2_CO_3_-3.14 synthesized by the excess Na_2_CO_3_ precipitation compared the best catalytic activity and stability, which could be reused for seven times without obvious deactivation. The DMC yield was as high as 69.97% at 68 °C. The transesterification reaction could be separated into two steps, and the samples obtained by NaOH precipitant exhibited better ring-opening capability, while the catalysts acquired by Na_2_CO_3_ precipitant displayed superior transesterification ability. The structure-performance relationship was evaluated by multiple characterization methods. The results indicated that the as-synthesized catalyst derived from dried precursors with more crystalline magnesium carbonate was favorable for the promotion of DMC yield, and MgO-P-Na_2_CO_3_-3.14 with more Mg-O pairs, which were the active center for the transesterification of 2-hydroxyethyl methyl carbonate (HEMC) intermediate with methanol, resulted in more moderately basic sites left that was in accordance with the DMC yield variation. MgO-P-Na_2_CO_3_-3.14 with greater BET surface area and mesopore volume, relative low surface oxygen content and larger moderately basic sites amount compared the excellent activity in DMC synthesis.

## Introduction

Dimethyl carbonate (DMC), which is the simplest dialkyl carbonates^1^, is widely applied in the chemical industry owing to its unique physicochemical properties^2^. It is an important intermediate for the synthesis of organic compounds and as an alkylating agent that can replace highly toxic dimethyl sulfate^3,4^. Besides, DMC is one of important fuel oil additives that contributes in decreasing emissions of particulate matter, hydrocarbon, and carbon monoxide from diesel and gasoline engines^5,6^. In recent years, the high-purity DMC with high dielectric constant is mainly applied in the lithium battery electrolyte^7,8^. As well known that DMC can be synthesized from phosgene, methanol oxidative carbonylation, transesterification of ethylene/propylene carbonate with methanol (MeOH), and urea alcoholysis^9,10^. We consider that the process of ethylene carbonate (EC) transesterification with MeOH is eco-friendly and economical with glycol as the co-product, which is a bulk commodity and can be applied as an intermediate for the production of polyester fibers and antifreeze.

Currently, DMC is industrially produced from the transesterification route with sodium methoxide as the catalyst^11,12^. Although it displays high transesterification activity, sodium methoxide is extremely liable to deactivation and cannot be recycled. Besides, as a homogeneous catalyst, it is difficult to separate from the reaction system. H_2_O and CO_2_ should be introduced into the reaction system to generate sodium carbonate, which has low solubility in glycol, leading to the formation of strongly alkaline solid waste. These drawbacks greatly limit its large-scale industrial applications. Therefore, developing highly active heterogeneous catalysts for DMC synthesis is of great importance.

As one important type of heterogeneous alkaline catalysts, magnesium oxide (MgO)^13,14^ or magnesium-containing catalysts^15–21^ have been extensively reported for the transesterification of EC with MeOH. Unnikrishnan et al.^19^ disclosed that the introduction of La in Mg–Al oxides synthesized by a co-precipitation method could enhance the basic strength of as-prepared catalysts. There existed a linear correlation between the catalytic activity and basic strength, and higher basic strength led to higher catalytic performance. Jesús Gandara-Loe et al.^20^ prepared six different layered double hydroxides (LDHs) catalysts derived from different interlayer anions (SiO_4_^4−^ or CO_3_^2−^) and divalent cations (Mg^2+^, Zn^2+^ or Ni^2+^), proving that the catalysts containing Ni^2+^ exhibited better catalytic activity owing to their higher basicity, while the samples containing SiO_4_^4−^ displayed higher selectivity because of a lower acid sites amount. Xu et al.^21^ synthesized a series of g-C_3_N_4_ supported MgO catalysts with varied MgO content and found that the incorporation of MgO effectively enhanced the overall basicity of g-C_3_N_4_. However, the transesterification activity of above-mentioned catalysts was not high enough because the reactions were conducted at higher temperature (70 ~ 140 °C)^19–21^ as well as greater MeOH to EC molar ratio of 10/1. The industrial production of DMC was usually carried out from 63.6 to 75 °C via catalytic distillation technology with MeOH/EC less than 6/1. At this operating condition, the process is the most energy efficient. Hence, it is important to develop highly active MgO catalysts in order to process the transesterification reaction under mild conditions.

To date, numerous methods have been developed to prepare MgO catalysts employed in multiple reactions^22–30^, such as thermal decomposition^22^, combustion^23^, co-precipitation^24^, hydrothermal synthesis^25^, sol–gel technique^26^, chemical vapor deposition^27^, and so on. Li et al.^28^ made a series of MgO with nanosheet morphology by thermal decomposition and precipitation methods. The resulting MgO catalysts were used for the synthesis of diethyl carbonate from ethyl carbamate and ethanol, demonstrating that sodium carbonate as the precipitant showed higher catalytic activity, which was attributed to a large amount of medium basic β sites. Belelli et al.^29^ reported a hydrothermal method for MgO synthesis applied in catalytic glycerolysis of methyl oleate. The density functional theory (DFT) results indicated that strongly basic low coordination O^2−^ that located on corners or edges of the MgO surface took part in kinetically relevant steps of the reaction. Sodium carbonate and a certain amount of sodium silicate were simultaneously reported as the precipitants to synthesize MgO for the removal of Congo red dye in aqueous solution, indicating that the capacity of adsorption was relevant to the surface base properties and the added amount of sodium silicate^30^.

In this work, a series of MgO catalysts were seriously prepared from hydration synthesis, thermal decomposition, combustion, sol–gel and co-precipitation methods. The effects of different types and concentrations of precipitants on the structural characteristics of MgO catalysts and the corresponding catalytic activity of EC transesterification with methanol to DMC were investigated and discussed in detail. The structure-performance relationship was assessed via detailed characterizations, including XRD, FT-IR, TG–DTA, SEM, N_2_ adsorption–desorption, CO_2_-TPD, XPS, TEM and HR-TEM technologies. Special attention was paid to MgO-P-Na_2_CO_3_-3.14 catalyst which displayed superior catalytic performance and stability due to greater BET surface area and mesopore volume, lower surface oxygen content as well as larger amount of moderately basic sites.

## Methods

### Materials

All the reagents were purchased from Aladdin Industrial Corporation, and were used directly without further purification.

### Preparation of catalysts

#### Direct thermal decomposition methods

Magnesium hydroxide (Mg(OH)_2_), basic magnesium carbonate (Mg_2_(OH)_2_CO_3_), magnesium nitrate hexahydrate (Mg(NO_3_)_2_•6H_2_O), magnesium citrate (Mg_3_(C_6_H_5_O_7_)_2_•9H_2_O), magnesium acetate (Mg(CH_3_COO)_2_•4H_2_O), and magnesium carbonate (MgCO_3_) were calcined in air at 500 °C for 3 h. The obtained references were noted as MgO-T-Mg(OH)_2_, MgO-T-Mg_2_(OH)_2_CO_3_, MgO-T-Mg(NO_3_)_2_, MgO-T-Mg_3_(C_6_H_5_O_7_)_2_, MgO-T-Mg(CH_3_COO)_2_ and MgO-T-MgCO_3_, respectively.

#### Co-precipitation methods

In a typical experiment, 0.4 mol Mg(NO_3_)_2_•6H_2_O was dissolved in 250 mL of deionized water to obtain solution A, while solution B contained different types and concentrations of alkali. The solution A and B were simultaneously dropped into 1000 mL of deionized water under vigorous stirring at 60 °C and a pH value of 10. The slurry was continuously stirred for 0.5 h after precipitation and then aged at room temperature for 20 h. The slurry was filtrated and washed with deionized water for eight times until there was no Na^+^ or K^+^ in the filtrate. The resulting precipitates were dried at 120 °C for 15 h, noted as MgO-P-KOH-Pre, MgO-P-K_2_CO_3_-Pre, MgO-P-NH_3_•H_2_O-Pre, MgO-P-NaOH-Pre, MgO-P-Na_2_CO_3_-Pre, and MgO-P-NaOH + Na_2_CO_3_-Pre, respectively. Afterwards, the precursors were calcined in air at 500 °C for 3 h. The obtained MgO catalysts were labeled as MgO-P-KOH, MgO-P-K_2_CO_3_, MgO-P-NH_3_•H_2_O, MgO-P-NaOH, MgO-P-Na_2_CO_3_, and MgO-P-NaOH + Na_2_CO_3_ (mixtures of NaOH and Na_2_CO_3_), respectively. The concentration of KOH, K_2_CO_3_, and NH_3_•H_2_O was 3 mol/L, 3 mol/L and 25–28 wt%, respectively. When single NaOH or Na_2_CO_3_ was applied as the precipitant, the concentration of NaOH or Na_2_CO_3_ was varied in order to investigate the influence of precipitant concentration on the structural properties and corresponding catalytic activity of MgO and the obtained catalysts were marked as MgO-P-NaOH-m and MgO-P-Na_2_CO_3_-n, respectively. Using NaOH and Na_2_CO_3_ mixtures as precipitants, the concentration ratios of NaOH and Na_2_CO_3_ were adjusted between 3/1 and 1/3 and the acquired samples were noted as MgO-P-NaOH + Na_2_CO_3_-m/n. In all situations, “m” represented the concentration of NaOH and “n” stood for the concentration of Na_2_CO_3_.

#### Other preparation methods

The hydration, citric acid-assisted combustion, and urea hydrolysis methods were applied according to the reported literatures^31–34^. The dried precursors were calcined in air at 500 °C for 3 h to obtain the target catalysts, were marked as MgO-hydration, MgO-C-C_6_H_8_O_7_, MgO-S-CH_3_COOH, and MgO-S-CO(NH_2_)_2_, respectively.

### Catalysts characterization

The powder X-ray diffraction (XRD) was conducted on a Bruker D8 advance diffractometer, equipped with a monochromatic Cu Kα (λ = 0.15406 nm) radiation. The scanning angle (2*θ*) was in the range from 10° to 90° with a scanning speed of 2°/min at 40 kV and 40 mA.

Fourier transform infrared (FT-IR) spectrum was acquired in the scope from 400 to 4000 cm^−1^ under air atmosphere in NEXUS 470 instrument.

The thermal decomposition behaviors of the dried precursors were carried out by thermogravimetric and differential thermal analysis (TG–DTA, STA 449C Jupiter, NETZSCH). The temperature was linearly increased from 30 to 900 °C at a heating rate of 20 °C /min in air atmosphere.

The surface morphology was studied by scanning electron microscopy (SEM, SIGMA 300). The samples were first coated with a platinum layer via an auto fine coater and then transferred into the SEM chamber.

The specific surface area was tested by nitrogen adsorption–desorption at 77 K on an Autosorb iQ Station 1 instrument. Catalysts were degassed at 300 °C before the isotherm was acquired. The surface area was calculated according to the Brunauer–Emmett–Teller (BET) method.

The basic properties of MgO catalysts were studied by temperature programmed desorption of carbon dioxide (CO_2_-TPD) using an AutoChem II automated adsorption instrument. About 50 mg sample was first degassed at 500 °C in He gas of 30 mL/min flow rate for 1 h and then cooled to 50 °C. Subsequently, the CO_2_ gas of 30 mL/min flow rate was introduced and absorbed for 1 h. The physically adsorbed CO_2_ was removed by He gas of 30 mL/min flow rate for 0.5 h. Finally, the temperature was linearly enhanced from 50 to 500 °C at a heating rate of 10 °C /min. The amount of desorbed CO_2_ was detected by a thermal conductivity detector (TCD).

X-ray photoelectron spectroscopy (XPS, Thermo ESCALAB 250 Xi, AlKα radiation) analysis was used to study the chemical composition and state on the catalysts surface. C 1 s line (284.6 eV) was employed as a reference to calibrate the binding energy (BE).

The transmission electron microscopy (TEM) and high-resolution transmission electron microscopy (HR-TEM) were obtained on a FEI-TALOS F200 (200 kV) instrument. The power was first dispersed in ethanol and then a drop of the suspension was put on a copper grid.

### Catalytic activity tests

The transesterification of EC with MeOH was conducted in a three-neck round-bottom flask of 250 mL, equipped with a heating jacket, a condenser, and a thermometer. The molar ratio of MeOH/EC was fixed at 6/1, while the catalyst amount was controlled at 3 wt% in terms of total mass of EC and MeOH. The reaction was performed at 68 °C for 0.5 h and then quickly cooled to room temperature. For the reusability study, the liquid products were separated by a rotary evaporator at 155–160 °C and − 0.1 MPa for 30 min. Thereafter, the left catalyst was reused at the same reaction conditions.

The as-prepared MgO_-P-Na2CO3-3.14_ catalyst was also tested in a continuous fixed-bed reactor. In a typical experiment, 1.0 g catalyst was placed in the middle of reactor with quartz wool to hold the catalyst bed. The reaction was conducted at atmospheric pressure and reaction temperature of 75–77 °C. The mixed liquid of EC and methanol with MeOH/EC molar ratio of 6/1 was introduced on the top of reactor by a high-pressure pump. The weight hourly space velocity (WHSV) was fixed at 5.0 h^−1^. The liquid products were condensed and collected in a cold trap.

The mixtures were filtrated and the liquid products were analyzed by using a gas chromatograph (GC-2010 pro) equipped with a HP-FFAP capillary column connected to a flame ionization detector (FID). The conversion of EC and the selectivity and yield of products were calculated as follows:

EC Conv. = 100 × (n _DMC_ + n _HEMC_ + n _DHEMC_) / (n _DMC_ + n _HEMC_ + n _DHEMC_ + n _EC left_).

DMC Sel. = 100 × n _DMC_ / (n _DMC_ + n _HEMC_ + n _DHEMC_).

HEMC Sel. = 100 × n _HEMC_ / (n _DMC_ + n _HEMC_ + n _DHEMC_).

DHEMC Sel. = 100 × n _DHEMC_ / (n _DMC_ + n _HEMC_ + n _DHEMC_).

DMC Yield = EC Conv. × DMC Sel.

Where the suffix “HEMC” and “DHEMC” represented 2-hydroxyethyl methyl carbonate and Di-2-hydroxyethyl methyl carbonate, which were two intermediates formed during the transesterification reaction. “n _DMC_”, “n _HEMC_”, and “n _DHEMC_” represented the generated molar numbers of DMC, HEMC, and DHEMC, respectively. “n _EC left_” stood for the molar numbers of unreacted EC after reaction.

The turnover frequency (TOF) was calculated using the following equations:

TOF_1_ = (n _EC_ × EC Conv.) / (n _MgO_ × t).

TOF_2_ = (n _EC_ × DMC Yield) / (n _MgO_ × t).

TOF_total_ = TOF_1_ + TOF_2_.

Where “TOF_1_” represented the turnover frequency of first step, which was associated with ring-opening capability of EC, leading to the generation of HEMC intermediate. “TOF_2_” stood for the turnover frequency of second step, which was related to the esterification ability of HEMC with methanol. “TOF_total_” was the total turnover frequency. “n _EC_”, “EC Conv.” and “DMC Yield” represented the molar number of added EC at initial reaction stage, the EC conversion and the DMC yield, respectively. “n _MgO_” was the molar number of MgO catalyst, while “t” was the reaction time.

## Results and discussion

### Catalytic activity measurements

#### Effect of catalysts prepared by different methods

In this section, different methods were applied for MgO synthesis in order to select optimum catalysts for transesterification and the results are summarized in Table 1. When the reaction was carried out without any catalyst (entry 1), EC conversion was as low as 10.13% with only 0.27% selectivity and 0.03% yield of DMC, indicating that EC was mainly converted to the intermediates, which were 2-hydroxyethyl methyl carbonate (HEMC) and Di-2-hydroxyethyl methyl carbonate (DHEMC), just by heating in the absence of the catalyst.

**Table 1:** The effect of different MgO preparation methods on transesterification efficiency.

Entry	Catalysts	EC Conv. (%)	DMC Sel. (%)	HEMC Sel. (%)	DHEMC Sel (%)	DMC Yield (%)	TOF_1_ (h^−1^)	TOF_2_ (h^−1^)	TOF_total_ (h^−1^)
1	No catalyst	10.13	0.27	99.18	0.55	0.03	0.96	0.003	0.96
2	MgO-commercial^a^	51.30	4.85	93.27	1.88	2.49	4.89	0.24	5.13
3	MgO-hydration	55.20	6.34	93.01	0.65	3.50	5.26	0.33	5.59
4	MgO-T-Mg(OH)_2_	50.03	9.19	88.82	1.99	4.60	4.76	0.44	5.20
5	MgO-T-Mg_2_(OH)_2_CO_3_	35.25	15.97	83.97	0.06	5.63	3.36	0.54	3.90
6	MgO-T-Mg(NO_3_)_2_	28.01	21.47	76.18	2.35	6.01	2.67	0.57	3.24
7	MgO-T-Mg_3_(C_6_H_5_O_7_)_2_	45.98	9.20	89.73	1.07	4.23	4.38	0.40	4.78
8	MgO-C-C_6_H_8_O_7_	44.44	12.67	86.69	0.64	5.63	4.23	0.54	4.77
9	MgO-T-Mg(CH_3_COO)_2_	24.82	24.21	73.45	2.34	6.01	2.36	0.57	2.93
10	MgO-S-CH_3_COOH	33.52	23.18	75.60	1.22	7.77	3.19	0.74	3.93
11	MgO-T-MgCO_3_	31.09	27.89	69.07	3.04	8.67	2.96	0.83	3.79
12	MgO-S-CO(NH_2_)_2_	55.04	14.23	84.36	1.41	7.83	5.24	0.75	5.99
13	MgO-P-NH_3_•H_2_O -25%	54.81	16.11	81.74	2.15	8.83	5.22	0.84	6.06
14	MgO-P-KOH-3	54.94	16.38	82.51	1.11	9.00	5.23	0.86	6.09
15	MgO-P-NaOH-3	55.29	18.81	79.01	2.18	10.40	5.26	0.99	6.25
16	MgO-P-K_2_CO_3_-3	45.05	39.84	57.01	3.15	17.95	4.29	1.71	6.00
17	MgO-P-Na_2_CO_3_-3	45.15	51.92	44.71	3.37	23.44	4.30	2.23	6.53

Reaction condition: molar ratio of EC/MeOH = 1/6, catalyst amount = 3 wt%, reaction temperature = 68 °C, reaction time = 0.5 h.

^a^The commercial MgO was calcined at 500 °C for 3 h before reaction.

Using commercial MgO as the catalyst (entry 2), EC conversion was promoted to 51.30% with 4.85% DMC selectivity and 2.49% DMC yield. When it was treated by deionized water (entry 3), both EC conversion and DMC selectivity of MgO-hydration clearly increased to 55.20% and 6.34% due to the improvement of surface area, which was supported by Ferretti et al.^32^. MgO catalysts prepared by the thermal decomposition of magnesium salts exhibited higher yield of DMC as compared with those of commercial MgO as well as MgO obtained from hydration treatment. Among them, MgO-T-Mg(OH)_2_ (entry 4) derived from Mg(OH)_2_ displayed the highest EC conversion about 50.03% but very low DMC yield around 4.60%. MgO-T-MgCO_3_ (entry 11) formed from MgCO_3_ showed the greatest DMC selectivity and yield, which were 27.89% and 8.67%, respectively. The DMC yield (5.63%) of MgO-T-Mg_2_(OH)_2_CO_3_ (entry 5) obtained from Mg_2_(OH)_2_CO_3_ was between those from MgO-T-Mg(OH)_2_ and MgO-T-MgCO_3_. Whereas MgO-T-Mg(NO_3_)_2_ (entry 6) coming from Mg(NO_3_)_2_ exhibited higher DMC yield than those of MgO-T-Mg(OH)_2_ and MgO-T-Mg_2_(OH)_2_CO_3_. The DMC yield of MgO catalysts prepared by direct thermal decomposition of different inorganic magnesium salts increased in the order of MgO-T-Mg(OH)_2_ (4.60%) < MgO-T-Mg_2_(OH)_2_CO_3_ (5.63%) < MgO-T-Mg(NO_3_)_2_ (6.01%) < MgO-T-MgCO_3_ (8.67%). As for MgO catalysts synthesized by thermal decomposition with organic magnesium salts, MgO-T-Mg_3_(C_6_H_5_O_7_)_2_ (entry 7) arose from Mg_3_(C_6_H_5_O_7_)_2_ exhibited lower DMC yield (4.23%) than that (6.01%) of MgO-T-Mg(CH_3_COO)_2_ (entry 9) derived from Mg(CH_3_COO)_2_•4H_2_O.

MgO-C-C_6_H_8_O_7_ (entry 8) prepared by the citric acid-assisted low temperature combustion method exhibited higher DMC yield of 5.63% than that of MgO-T-Mg_3_(C_6_H_5_O_7_)_2_ (entry 7). MgO-S-CH_3_COOH (entry 10) synthesized by a sol–gel method with acetic acid displayed much higher EC conversion 33.52% and DMC yield (7.77%) than those of MgO-T-Mg(CH_3_COO)_2_ (entry 9). MgO-S-CO(NH_2_)_2_ (entry 12) acquired from urea hydrolysis showed greater DMC yield (7.83%) than those of MgO samples received from the combustion method as well as the sol–gel method.

As shown in Table 1, MgO catalysts prepared by a co-precipitation method compared much higher DMC yield than those of MgO acquired by other methods, indicating excellent catalytic activity. Besides, the types of precipitants had significant effects on EC conversion and DMC selectivity. When 3.0 mol/L NaOH was used as the precipitant, the EC conversion of MgO-P-NaOH (entry 15) was as high as 55.29%, which was similar to that of MgO prepared by 3.0 mol/L KOH (entry 14) or 25 wt% NH_3_•H_2_O (entry 13). When the same concentration of K_2_CO_3_ (entry 16) and Na_2_CO_3_ (entry 17) were employed as the precipitant, although the EC conversion decreased by 10% level, the DMC selectivity and yield had significant increasement. Especially for MgO-P-Na_2_CO_3_-3, it displayed about 23.44% DMC yield which was nearly 2.3 times larger than that MgO-P-NaOH-3. Among all the precipitated catalysts, MgO-P-Na_2_CO_3_-3 obtained from Na_2_CO_3_ showed the highest DMC yield.

The transesterification of EC with methanol could be separated into two steps. The first step was ring-opening reaction of ethylene carbonate, resulting in the formation of HEMC intermediate (reaction (1)). The second step was the transesterification of HEMC with methanol, leading to the generation of final product DMC (reaction (2)). In order to further compare the catalytic activity of MgO catalysts prepared by different methods, the turnover frequency of first step (TOF_1_) and second step (TOF_2_) were also calculated and exhibited in Table 1. It was obvious that MgO-hydration and MgO-P-NaOH-3 displayed the highest TOF_1_ value (5.26 h^−1^), followed by MgO-S-CO(NH_2_)_2_ (5.24 h^−1^), MgO-P-KOH-3 (5.23 h^−1^) and MgO-P-NH_3_•H_2_O-25% (5.22 h^−1^). However, the TOF_2_ value of MgO-P-NaOH-3 was as low as 0.99 h^−1^. Above results implied that the MgO sample prepared by 3.0 mol/L NaOH showed superior ring-opening capability but poor esterification ability, leading to higher EC conversion (55.29%) and HEMC selectivity (79.01%) but lower DMC selectivity (18.81%). In contrast, MgO-P-Na_2_CO_3_-3 showed the highest TOF_2_ value (2.23 h^−1^), although the TOF_1_ value was not high (4.30 h^−1^). This indicated that MgO catalyst obtained by 3.0 mol/L Na_2_CO_3_ exhibited outstanding esterification capability, resulting in the increased DMC selectivity (51.92%). Among all the catalyst preparation methods, MgO obtained by co-precipitation method displayed much higher TOF_total_ values than those of other methods. Therefore, it was concluded that MgO samples derived from co-precipitation showed better catalytic performance. (1)
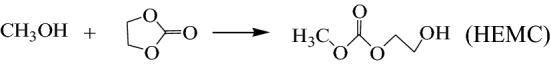


(2)
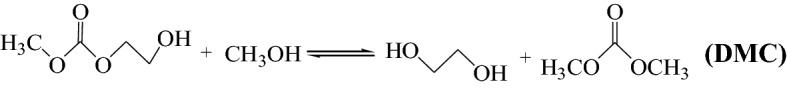


(3)
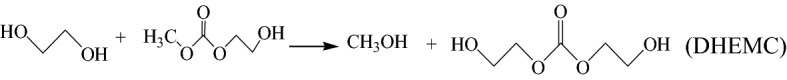


In conclusion, the DMC yield of MgO catalysts followed the order of no catalyst < MgO-commercial < MgO-hydration < MgO-T-Mg_3_(C_6_H_5_O_7_)_2_ < MgO-T-Mg(OH)_2_ < MgO-T-Mg_2_(OH)_2_CO_3_ ≈ MgO-C-C_6_H_8_O_7_ < MgO-T-Mg(NO_3_)_2_ ≈ MgO-T-Mg(CH_3_COO)_2_ < MgO-S-CH_3_COOH < MgO-S-CO(NH_2_)_2_ < MgO-T-MgCO_3_ < MgO-P-NH_3_•H_2_O-25% < MgO-P-KOH-3 < MgO-P-NaOH-3 < MgO-P-K_2_CO_3_-3 < MgO-P-Na_2_CO_3_-3. MgO prepared by NaOH or Na_2_CO_3_ were selected as target catalysts and the effects of single NaOH or Na_2_CO_3_ concentration as well as the concentration ratios of NaOH/Na_2_CO_3_ on the structural properties and corresponding catalytic activity were systematically investigated in the following sections.

#### Effect of different precipitant concentrations, types, and reaction parameters

**Figure 1:**
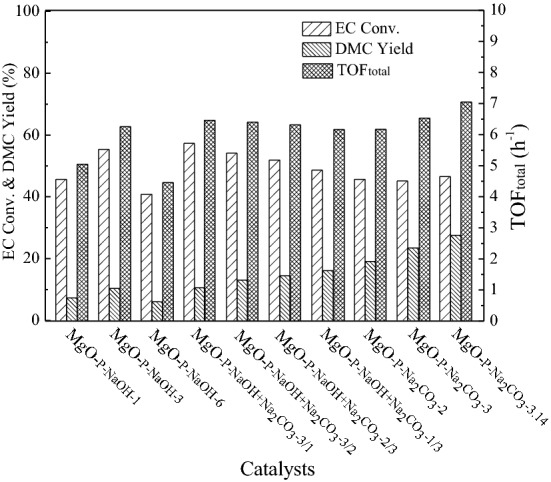
The effect of different precipitant concentrations and types on the catalytic activity. Reaction conditions: EC/MeOH = 1/6, catalyst amount = 3 wt%, reaction temperature = 68 °C, reaction time = 0.5 h.

The effect of different precipitant concentrations and types on the catalytic activity of as-prepared MgO is shown in Fig. 1. Using 1 mol/L NaOH aqueous solution as the precipitant, the EC conversion and DMC yield were 45.64% and 7.34%, respectively. With enhancing the concentration from 1 to 3 mol/L, the conversion of EC and the yield of DMC clearly increased to 55.29% and 10.40%. Further increasing the concentration to 6 mol/L, both the EC conversion and DMC yield obviously dropped to 40.77% and 6.05%, which was even lower than that from using 1 mol/L NaOH. When 2 mol/L Na_2_CO_3_ aqueous solution was employed as the precipitant, the conversion of EC was 45.62% with 19.14% DMC yield. By gradually improving the concentration from 2 to 3 mol/L, the EC conversion kept nearly unchanged, while the DMC yield rapidly increased to 23.44%, which was twofold larger than that of MgO-P-NaOH-3 prepared with the same concentration of NaOH aqueous solution. Further enhancing the concentration to 3.14 mol/L (saturated Na_2_CO_3_ aqueous solution), the conversion of EC remained the same, yet the yield of DMC had a slight increase from 23.44% to 27.51%. Using NaOH and Na_2_CO_3_ mixtures as precipitants, it can be seen that the EC conversion exhibited a linear increase from 48.61% to 57.29%, whereas the DMC yield displayed a rapid decrease from 16.11% to 10.55% with enhancing the NaOH/Na_2_CO_3_ concentration ratio from 1/3 to 3/1. We considered that high EC conversion was attributed to the great ring-opening rate, while high DMC yield corresponded to the high rate of transesterification efficiency. Therefore, it was deduced that MgO obtained by optimum concentration of NaOH showed excellent ring-opening ability and MgO prepared by saturated concentration of Na_2_CO_3_ compared superior transesterification capability.

In order to further evaluate the effect of different precipitant concentrations and types on the transesterification of EC with methanol, the total turnover frequency (TOF_total_) was also calculated and displayed in Fig. 1. The TOF_total_ of MgO-_P-NaOH_^−1^ was 5.05 h^−1^. By enhancing NaOH concentration from 1.0 to 3.0 mol/L, the TOF_total_ of MgO-_P-NaOH-3_ clearly increased to 6.26 h^−1^. Further increasing NaOH concentration (6.0 mol/L) led to the decreased TOF_total_ value. By introducing Na_2_CO_3_ in precipitate, the TOF_total_ of MgO-P-NaOH + Na_2_CO_3_-3/1, MgO-P-NaOH + Na_2_CO_3_-3/2, MgO-P-NaOH + Na_2_CO_3_-2/3 and MgO-P-NaOH + Na_2_CO_3_^−1^/3 obviously increased to 6.46, 6.40, 6.32 and 6.16 h^−1^, respectively. The TOF_total_ value further enhanced when single Na_2_CO_3_ was used as a precipitate, especially for MgO-P-Na_2_CO_3_-3.14 prepared with saturated Na_2_CO_3_ aqueous solution which exhibited the highest TOF_total_ (7.05 h^−1^) among all the catalysts. Therefore, the TOF_total_ further indicated that the MgO sample obtained via excess Na_2_CO_3_ precipitation exhibited the best catalytic activity.

**Figure 2:**
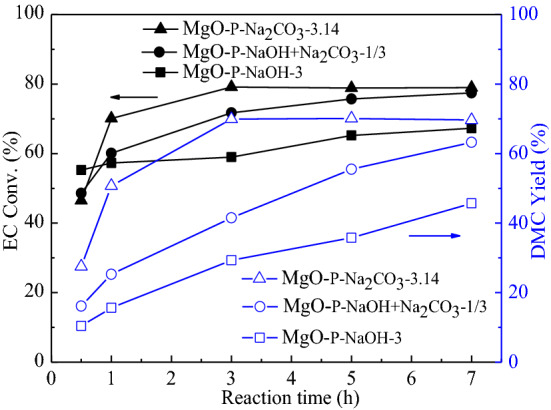
The effect of reaction time on the transesterification reaction. Reaction conditions: EC/MeOH = 1/6, catalyst amount = 3 wt%, reaction temperature = 68 °C.

The influence of reaction time changing from 0.5 to 7 h of MgO-_P-NaOH-3_, MgO-P-Na_2_CO_3_-3.14, and MgO-P-NaOH + Na_2_CO_3_-1/3 on transesterification efficiency is displayed in Fig. 2. When the reaction time was 0.5 h, MgO-_P-NaOH-3_ exhibited the highest EC conversion and the lowest DMC yield, indicating advanced ring-opening capability but poor transesterification ability. In contrast, MgO-P-Na_2_CO_3_-3.14 displayed the lowest EC conversion and the greatest DMC yield, revealing excellent transesterification capability but inferior ring-opening ability. These results were in good agreement with previous discussion. With expanding reaction time from 0.5 to 1 h, the EC conversion of MgO-P-Na_2_CO_3_-3.14 and MgO-P-NaOH + Na_2_CO_3_-1/3 catalysts underwent a linear increase from 46.54% to 70.12% and from 48.61% to 60.12%, respectively. However, the EC conversion of MgO-_P-NaOH-3_ only had a slight increase from 55.29% to 57.28%. For these three catalysts, the DMC yield all went through a rapid growth with enhancing reaction time to 1 h. By increasing reaction time from 1 to 3 h, both EC conversion and DMC yield of MgO-P-Na_2_CO_3_-3.14 clearly increased and were as high as 79.14% and 69.97%, respectively. Further increasing reaction time to 7 h, both of them kept nearly unchanged, demonstrating that the transesterification of EC with MeOH attained the chemical equilibrium point at 3 h. However, it was obvious that the EC conversion and DMC yield of MgO-_P-NaOH-3_ and MgO-P-NaOH + Na_2_CO_3_-1/3 catalysts continuously increased and did not reach the equilibrium until 7 h. Hence, it could be concluded that MgO-P-Na_2_CO_3_-3.14 exhibited higher transesterification efficiency as compared with that of MgO-_P-NaOH-3_ and MgO-P-NaOH + Na_2_CO_3_-1/3.

It was obvious that the types and concentrations of precipitants had significant influences on EC conversion and DMC yield. Using single NaOH aqueous solution as the precipitant, the as-prepared MgO catalyst displayed higher EC conversion. However, employing single Na_2_CO_3_ aqueous solution as the precipitant, the as-synthesized MgO sample exhibited greater DMC yield and the value was clearly promoted with gradually increasing the concentration of Na_2_CO_3_. Using mixtures of NaOH and Na_2_CO_3_ as precipitants, the EC conversion displayed a rapid increase while the DMC yield exhibited a linear decrease with enhancing the concentration ratio of NaOH/Na_2_CO_3_. Among all the catalysts, MgO-P-Na_2_CO_3_-3.14 prepared by the saturated Na_2_CO_3_ aqueous solution (3.14 mol/L) compared the best catalytic activity. The transesterification reaction reached chemical equilibrium at 3 h with 79.14% conversion of EC and 69.97% yield of DMC. In the following sections, the detailed characterization methods were systematically investigated and discussed in order to reveal the relationship between the physicochemical properties and the corresponding catalytic performance.

### Catalyst characterization

#### Crystal structure analysis of the dried precursors and catalysts

**Figure 3:**
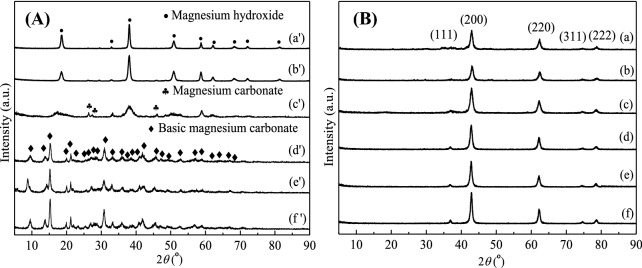
XRD patterns of the dried precursors (**A**) and the calcined catalysts (**B**). (**A**): (a') MgO-P-NaOH-3-Pre, (b') MgO-P-NaOH-6-Pre, (c') MgO-P-NaOH + Na_2_CO_3_-3/1-Pre, (d') MgO-P-NaOH + Na_2_CO_3_-1/3-Pre, (e') MgO-P-Na_2_CO_3_-3-Pre, and (f') MgO-P-Na_2_CO_3_-3.14-Pre. (**B**): (a) MgO-P-NaOH-3, (b) MgO-P-NaOH-6, (c) MgO-P-NaOH + Na_2_CO_3_-3/1, (d) MgO-P-NaOH + Na_2_CO_3_-1/3, (e) MgO-P-Na_2_CO_3_-3, and (f) MgO-P-Na_2_CO_3_-3.14.

XRD patterns of the dried precursors prepared with different types and concentrations of precipitants are shown in Fig. 3A. It was clear that all the diffraction peaks of MgO-P-NaOH-3-Pre (a') and MgO-P-NaOH-6-Pre (b') were attributed to magnesium hydroxide (Mg(OH)_2_), of which the crystallinity reduced with the increased NaOH concentration. With the introduction of Na_2_CO_3_ in precipitant, the diffraction peaks of MgO-P-NaOH + Na_2_CO_3_-3/1-Pre (c') were indexed to the mixture of magnesium hydroxide and magnesium carbonate (MgCO_3_). However, the peaks of magnesium hydroxide were much broader than those of MgO-P-NaOH-3-Pre and MgO-P-NaOH-6-Pre, demonstrating the generation of smaller crystallite size and lower crystallinity of magnesium hydroxide. By continuously increasing Na_2_CO_3_ content in precipitant, the XRD patterns of MgO-P-NaOH + Na_2_CO_3_-1/3-Pre (d') exhibited one single phase, which was ascribed to basic magnesium carbonate (Mg_5_(CO_3_)_4_(OH)_2_•4H_2_O). When Na_2_CO_3_ was used as the exclusive precipitant, all diffraction peaks of the MgO-P- Na_2_CO_3_-3-Pre (e') and MgO-P- Na_2_CO_3_-3.14-Pre (f') were also derived from basic magnesium carbonate, of which the crystallinity gradually increased with enhancing Na_2_CO_3_ concentration.

Figure 3B displayed the XRD patterns of the calcined catalysts. All the samples exhibited diffraction peaks at 2θ of 36.94°, 42.92°, 62.32°, 74.70°, and 78.64°, which were attributed to (111), (200), (220), (311), and (222) planes of cubic MgO (PDF-#45–0946) with space group Fm-3 m (225). No other impurities were observed, indicating that the dried precursors completely decomposed to MgO after calcination at 500 °C. The crystallite sizes of MgO were calculated based on the diffraction peak with the strongest intensity at 2θ of 42.92° using Scherrer's formula. As summarized in Table 2, it was clear that the types and concentrations of precipitants had significant influences on the MgO crystallite sizes. The crystallite sizes variation was in the order of MgO-P-NaOH-6 < MgO-P-NaOH-3 < MgO-P-NaOH + Na_2_CO_3_-3/1 < MgO-P-NaOH + Na_2_CO_3_-1/3 < MgO-P-Na_2_CO_3_-3 < MgO-P-Na_2_CO_3_-3.14. It could be concluded that the same concentration of Na_2_CO_3_ precipitate compared much larger MgO crystallite sizes than that of NaOH. Of all the as-synthesized MgO catalysts, the crystallite sizes decreased with the enhanced NaOH concentration and increased with the promoted concentration of the Na_2_CO_3_ precipitate, of which saturated Na_2_CO_3_ solution displayed the largest MgO crystallite size.

**Table 2:** The characterization of various MgO catalysts.

Catalysts	Specific surface area^a^ (m^2^/g)	Total pore volume^b^ (cm^3^/g)	Average pore size^c^ (nm)	Crystallite sizes (nm)
MgO-P-NaOH-3	99	0.44	17.41	8.7
MgO-P-NaOH-6	73	0.32	17.38	7.6
MgO-P-NaOH + Na_2_CO_3_-3/1	104	0.48	18.53	10.4
MgO-P-NaOH + Na_2_CO_3_-1/3	137	0.88	23.66	11.7
MgO-P-Na_2_CO_3_-3	149	0.88	23.68	12.0
MgO-P-Na_2_CO_3_-3.14	162	0.97	23.91	13.1

^a^Calculated by Brunauer–Emmett–Teller (BET) method;

^b^Volume adsorbed at P/P_0_ = 0.99;

^c^Calculated based on BJH method.

#### Functional group analysis of the dried precursors

**Figure 4:**
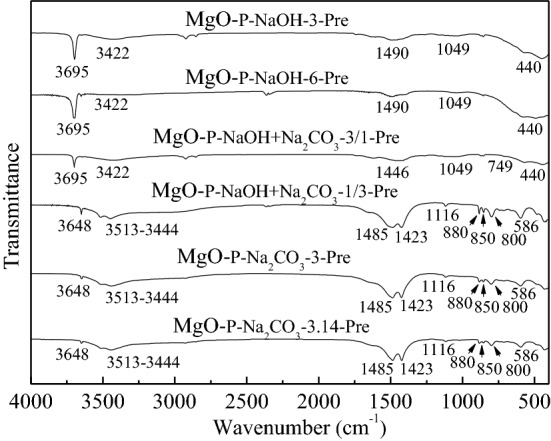
FTIR spectra of the dried precursors.

FTIR analysis was used to investigate the functional groups in the dried precursors and the spectra are shown in Fig. 4. For MgO-_P-NaOH-3-Pre_ and MgO-_P-NaOH-6-Pre_, there existed five adsorption peaks in the wavenumber range from 400 to 4000 cm^−1^. The first sharp peak with strong intensity at about 3695 cm^−1^ was attributed to the stretching vibration of hydroxide ion (OH^−^)^35^. The second and fourth broad peaks at around 3422 and 1049 cm^−1^ were associated with the stretching and bending vibrations of hydroxyl group (-OH) derived from water molecules^36,37^. The third wide peak at about 1490 cm^−1^ might be ascribed to the adsorbed CO_2_ molecules on the surface of precursor^38^. The last peak at 440 cm^−1^ was corresponded to the stretching vibration of Mg-O band in magnesium hydroxide^39^. Therefore, the FTIR analysis clearly indicated that the dried precursor obtained by single NaOH precipitate was only magnesium hydroxide, which was consistent with the former XRD analysis.

With the addition of Na_2_CO_3_ in the precipitate, MgO-P-NaOH + Na_2_CO_3_-3/1-pre exhibited similar adsorption peaks as those of MgO-_P-NaOH-3-Pre_ and MgO-_P-NaOH-6-Pre_. The only two differences were the first formation of broad peaks at about 1446 and 749 cm^−1^, which were derived from the asymmetric stretching vibration and in-plane bending vibration of carbonate (CO_3_^2−^)^40,41^, indicating that not only magnesium hydroxide but also magnesium carbonate appeared in MgO-P-NaOH + Na_2_CO_3_-3/1-pre. By increasing the content of Na_2_CO_3_ in precipitate, the FTIR spectra of MgO-P-NaOH + Na_2_CO_3_-1/3-pre occurred a great change. The adsorption peak at about 3648 cm^−1^ was assigned to the vibration of free –OH^42^. The peaks between 3513 and 3444 cm^−1^ were associated with the stretching vibration of -OH group, which was derived from crystal water. In addition, the carbonate asymmetric stretching vibration split into two adsorption peaks at 1485 and 1423 cm^−1^, while the bending vibration of carbonate divided into three adsorption peaks at 880, 850, and 800 cm^−1^. Furthermore, the peaks at 1116 and 586 cm^−1^ were first observed, which were attributed to the symmetric stretching vibration of carbonate as well as the Mg-O-Mg band vibration, respectively^37,43^. Above all of features were in good accordance with the characteristic adsorption of basic magnesium carbonate, indicating that MgO-P-NaOH + Na_2_CO_3_-1/3-pre was composed of basic magnesium carbonate phase. When single Na_2_CO_3_ was used as the precipitate, the FTIR spectra of MgO-P-Na_2_CO_3_-3-pre and MgO-P-Na_2_CO_3_-3.14-pre were nearly the same as those of MgO-P-NaOH + Na_2_CO_3_-1/3-pre, demonstrating that the main phase of the dried precursor formed by single Na_2_CO_3_ precipitate was also basic magnesium carbonate.

#### Thermal decomposition behaviors of the dried precursors

**Figure 5:**
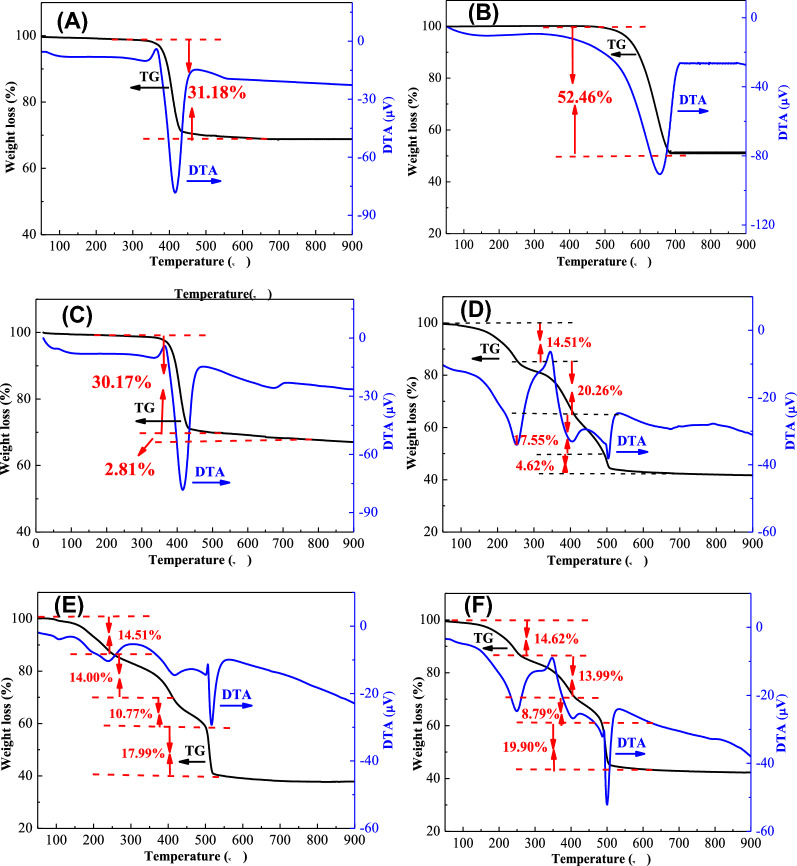
(**A**–**F**) TG–DTA curves of the dried precursors prepared by different types and concentrations of precipitants: (**A**) MgO-P-NaOH-3-Pre, (**B**) pure MgCO_3_, (**C**) MgO-P-NaOH + Na_2_CO_3_-3/1-Pre, (**D**) MgO-P-NaOH + Na_2_CO_3_-1/3-Pre, (**E**) MgO-P-Na_2_CO_3_-3-Pre, (**F**) MgO-P-Na_2_CO_3_-3.14-Pre.

The thermal decomposition behaviors of the dried precursors prepared by different types and concentrations of precipitants were studied by TG–DTA analysis and the results are compared in Fig. 5A–F. As displayed in Fig. 5A, it was clear that there existed only one endothermic peak at about 400 °C. The corresponding weight loss was 31.18 wt%, which was nearly consistent with that of the decomposition of magnesium hydroxide to produce magnesium oxide (100 × 18.0/58.0 = 31.0 wt%), indicating that the dried precursor obtained from single NaOH precipitate was composed of magnesium hydroxide phase. This result was in good agreement with the previous XRD and FTIR analysis.

Figure 5B shows the thermal decomposition curve of pure magnesium carbonate. There also existed only one endothermic peak at about 650 °C, which was significantly higher than that of magnesium hydroxide, revealing that the decomposition of magnesium carbonate was conducted only at high temperature. The total weight loss was 52.46 wt%, which was almost in accordance with the theoretical calculation value of magnesium carbonate decomposition to form magnesium oxide (100 × 44.0/84.3 = 52.2 wt%).

As exhibited in Fig. 5C, the DTA curve of MgO-P-NaOH + Na_2_CO_3_-3/1-Pre displayed two endothermic peaks. The first sharp peak at 400 °C with severe endotherm and an abrupt weight loss of 30.17% was derived from the decomposition of magnesium hydroxide. The second broad peak at 650 °C with mild endotherm and a gentle weight loss of 2.81% arose from the magnesium carbonate decomposition. Therefore, the TG–DTA analysis suggested that MgO prepared with the NaOH/Na_2_CO_3_ concentration ratio of 3/1 was constituted of the mixtures of magnesium hydroxide and a little magnesium carbonate. This finding was also in line with the former XRD analysis.

As shown in Fig. 5D–F, the DTA curves of MgO-P-NaOH + Na_2_CO_3_-1/3-Pre, MgO-P-Na_2_CO_3_-3-Pre and MgO-P-Na_2_CO_3_-3.14-Pre exhibited three endothermic peaks and one exothermic peak. The first peak at about 250 °C was ascribed to the basic magnesium carbonate dehydration^44^. Simultaneously, TG curve showed a gentle weight loss of 14.5–14.6 wt%. The second broad peak at around 400 °C with mild endotherm and a gentle weight loss was attributed to the dehydroxylation and decarbonation of basic magnesium carbonate^45^. The last sharp peak at about 500 °C with severe endotherm and a sudden weight loss came from the generation of magnesium oxide via further decomposition of basic magnesium carbonate^46^. A sharp exothermic peak at about 490 °C with mild exotherm and a rapid weight loss was derived from the crystallization of amorphous magnesium carbonate, followed by the decomposition of crystalline magnesium carbonate, which was supported by Dell et al.^47^ and Sawada et al.^48^, indicating that there existed small amount of amorphous magnesium carbonate when using Na_2_CO_3_ as precipitate. The total weight loss was around 57% for these dried precursors, which was almost consistent with the theoretical calculation value of the decomposition of basic magnesium carbonate to produce magnesium oxide (100 × (5 × 18 + 4 × 44)/466 = 57.1 wt%), demonstrating that MgO-P-NaOH + Na_2_CO_3_-1/3-Pre, MgO-P-Na_2_CO_3_-3-Pre and MgO-P-Na_2_CO_3_-3.14-Pre were composed of basic magnesium carbonate phase.

As displayed in Fig. 5D–F, the weight loss of the third step was in the order of MgO-P-NaOH + Na_2_CO_3_-1/3-Pre (17.55%) ˃ MgO-P-Na_2_CO_3_-3-Pre (10.77%) ˃ MgO-P- Na_2_CO_3_-3.14-Pre (8.79%). As stated above, the third sharp exothermic peak was associated with the variation of amorphous magnesium carbonate to crystalline state and the simultaneous decomposition of amorphous magnesium carbonate to MgO. Therefore, the greater content of amorphous magnesium carbonate led to the higher weight loss. Incorporation of the reaction results in Fig. 1A, MgO-P-Na_2_CO_3_-3.14 displayed the highest DMC yield (27.51%), followed by MgO-P-Na_2_CO_3_-3 (23.44%) and MgO-P-NaOH + Na_2_CO_3_-1/3 (16.11%). Hence, it could be deduced that the MgO catalysts derived from the dried precursors with less amorphous magnesium carbonate were favorable for DMC yield increasement. In another saying, MgO came from the decomposition of crystalline magnesium carbonate exhibited an enhanced activity.

Based on TG–DTA analysis, it was obviously seen that there was almost no weight loss for all the dried precursors except for pure magnesium carbonate when the temperature was higher than 500 °C, showing that all the dried precursors were decomposed to magnesium oxide completely after calculation in air at 500 °C. As reported by Janet et al.^49^, higher calcination temperature could result in the sinter and agglomeration of MgO particles, which was not beneficial for the generation of inherent porous structure. Hence, the calcination temperatures of these dried precursors were fixed at 500 °C in order to avoid the agglomeration of MgO particles.

#### Surface morphology analysis of catalysts

**Figure 6:**
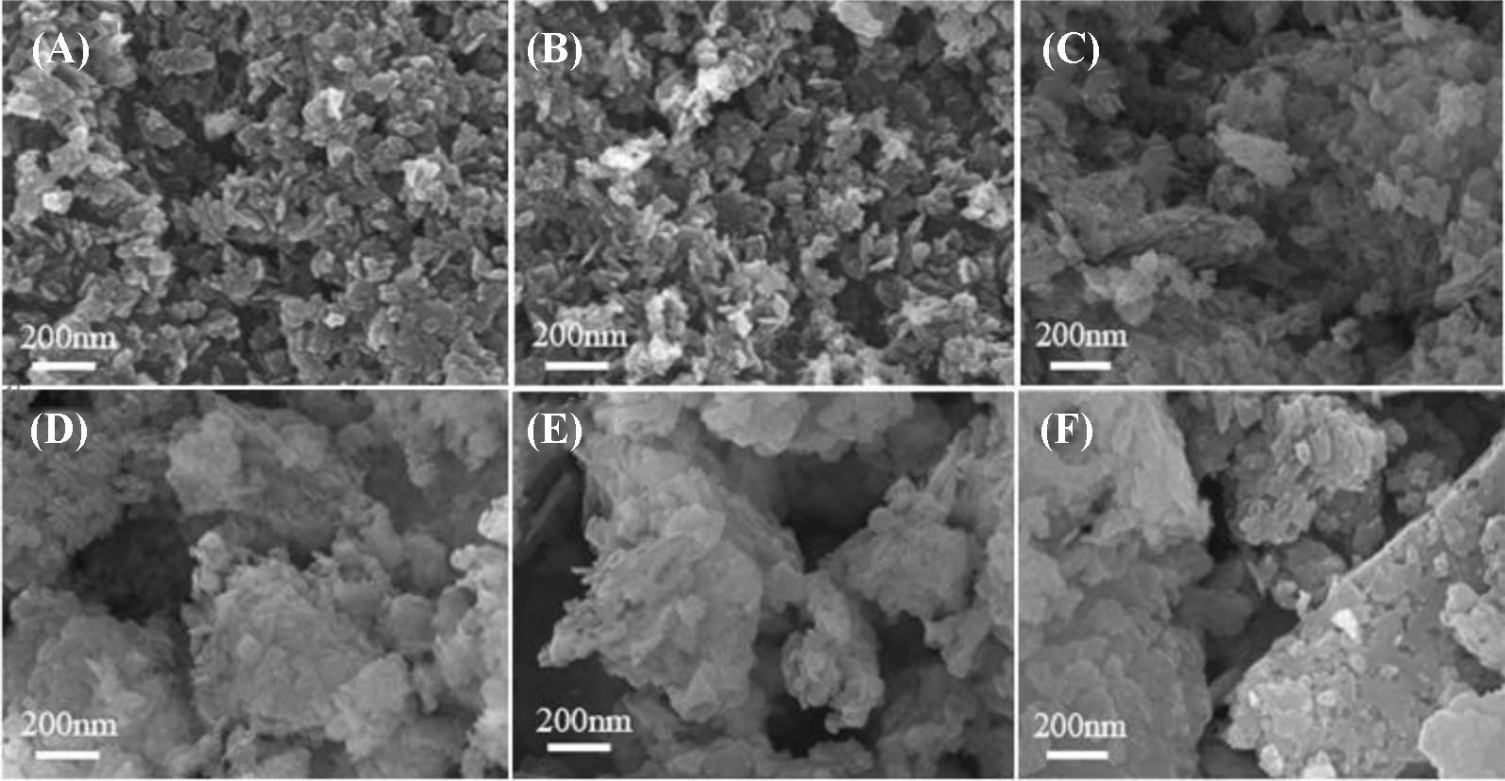
SEM images of (**A**) MgO-P-NaOH-3, (**B**) MgO-P-NaOH-6, (**C**) MgO-P-NaOH + Na_2_CO_3_-3/1, (**D**) MgO-P-NaOH + Na_2_CO_3_-1/3, (**E**) MgO-P-Na_2_CO_3_-3 and (**F**) MgO-P-Na_2_CO_3_-3.14.

SEM micrographs of different MgO catalysts are compared in Fig. 6A–F. All of samples displayed sheet-like morphologies. The particles of MgO-P-NaOH-3 and MgO-P-NaOH-6 were highly dispersed with uniform distribution. Numerous particles of MgO-P-NaOH + Na_2_CO_3_-1/3 and MgO-P-NaOH + Na_2_CO_3_-3/1 were gathered and the particle size was larger than that of MgO prepared by single NaOH precipitant. The bulk particles with irregular distribution were observed on MgO-P-Na_2_CO_3_-3 and MgO-P-Na_2_CO_3_-3.14. The variation trend of particle size was in the order of MgO-P-Na_2_CO_3_-3.14 > MgO-P-Na_2_CO_3_-3 ˃ MgO-P-NaOH + Na_2_CO_3_-1/3 > MgO-P-NaOH + Na_2_CO_3_-3/1 ˃ MgO-P-NaOH-3 > MgO-P-NaOH-6, which was in good agreement with that of the crystallite size calculated by Scherrer's formula (Table 2).

#### Pore structure and surface area analysis of catalysts

**Figure 7:**
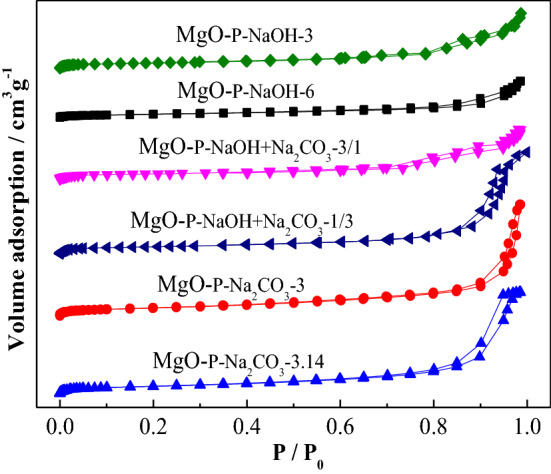
Nitrogen adsorption–desorption isotherms of different MgO catalysts.

Figure 7 presents the nitrogen adsorption–desorption isotherms of as-synthesized MgO, which were derived from different types and concentrations of precipitates. The curves displayed a typical IV isotherm including a hysteresis loop at a relative pressure greater than P/P_0_ = 0.6^50^, suggesting the presence of mesoporous structure of all the MgO catalysts. The hysteresis loop of MgO-P-NaOH-3 was similar to that of MgO-P-NaOH-6, indicating the existence of similar pore sizes. With the introduction of Na_2_CO_3_ in precipitant, the hysteresis loop of both MgO-P-NaOH + Na_2_CO_3_-3/1 and MgO-P-NaOH + Na_2_CO_3_-1/3 was obviously larger than that of MgO precipitated by single NaOH. More importantly, MgO obtained with single Na_2_CO_3_ exhibited much larger hysteresis loop than that of other samples, especially for MgO-P-Na_2_CO_3_-3.14 prepared via excess Na_2_CO_3_ precipitation. These findings demonstrated that the addition of Na_2_CO_3_ resulted in increased pore size and higher Na_2_CO_3_ concentration was favorable for obtaining larger pore size. The average pore size, which was calculated according to the BJH method and displayed in Table 2, was in good agreement with the above discussion and followed the order of MgO-P-Na_2_CO_3_-3.14 ˃ MgO-P-Na_2_CO_3_-3 ˃ MgO-P-NaOH + Na_2_CO_3_-1/3 ˃ MgO-P-NaOH + Na_2_CO_3_-3/1 ˃ MgO-P-NaOH-3 ≈ MgO-P-NaOH-6.

BET surface area and total pore volume of MgO-P-NaOH-3 were 99 m^2^/g and 0.44 cm^3^/g, respectively. By increasing NaOH concentration, both BET surface area and total pore volume of MgO-P-NaOH-6 decreased obviously. We considered that higher NaOH concentration led to partial amorphization of the precursor and the pore was blocked with amorphous magnesium hydroxide debris, resulting in the decreased BET surface area and pore volume. This phenomenon was also consistent with the previous XRD analysis (Fig. 3A) that the crystallinity of precursor reduced with the increased NaOH concentration. By introducing Na_2_CO_3_, BET surface area and total pore volume of MgO-P-NaOH + Na_2_CO_3_-1/3 and MgO-P-NaOH + Na_2_CO_3_-3/1 were much larger than those of MgO-P-NaOH-3 and MgO-P-NaOH-6. Using single Na_2_CO_3_ as the precipitant, both BET surface area and total pore volume were further promoted. Above results definitely proved that the addition of Na_2_CO_3_ led to the increase of surface area and pore volume, and higher Na_2_CO_3_ concentration resulted in the formation of highly porous structure of MgO. Incorporation of the reaction results in Fig. 1, it was obvious that the variation tendency of DMC yield was in accordance with that of the BET surface area as well as the average pore size, because the enlarged surface area and pore size contributed to improving the mass transfer efficiency of reactants. Of all the as-synthesized MgO catalysts, MgO-P-Na_2_CO_3_-3.14 displayed the largest BET surface area (162 m^2^/g), pore size (23.91 nm), and the highest DMC yield.

#### Basic properties of catalysts

**Figure 8:**
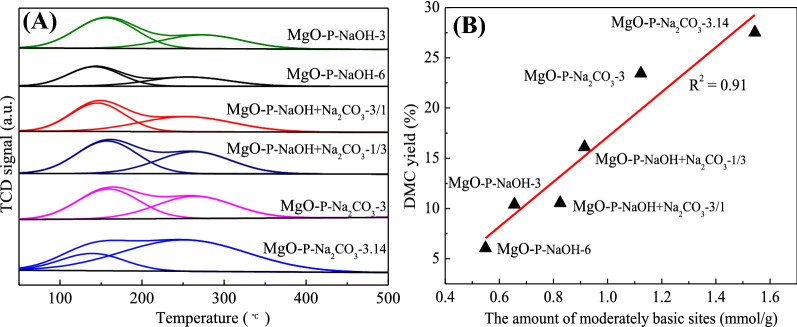
(**A**) CO_2_-TPD curves of different MgO catalysts. (**B**) The relationship between DMC yield and amount of moderately basic sites of various MgO samples.

CO_2_-TPD analysis is applied to investigate the surface basic properties of the as-prepared MgO samples and the TPD curves are compared in Fig. 8A. It was clear that all the catalysts displayed two desorption peaks of CO_2_ in the temperature range from 50 to 500 °C. The low temperature desorption peak (50–160 °C) was derived from the weakly basic sites, which corresponded to the hydroxyl group adsorbed on the catalyst surface, while the peak with high desorption temperature (160–400 °C) was attributed to the moderately basic sites, which was associated with Mg–O pairs^28,51,52^.

The number of basic sites were calculated based on the desorption of CO_2_ and summarized in Table 3. The number of weakly basic sites and moderately basic sites of MgO-P-NaOH-3 were 1.403 and 0.656 mmol/g, respectively. For MgO-P-NaOH-6 prepared with high concentration of NaOH, both weakly and moderately basic sites decreased obviously. By introducing Na_2_CO_3_ in precipitant, the number of weakly and moderately basic sites increased evidently as compared with those of MgO prepared by single NaOH. Moreover, when single Na_2_CO_3_ was used as the precipitant, the number of moderately basic sites further increased and a maximum value (1.544 mmol/g) was obtained over MgO-P-Na_2_CO_3_-3.14 through excess Na_2_CO_3_ precipitation. The addition of Na_2_CO_3_ was conducive to the increase of BET surface area (Table 2). As reported by Gao et al.^53^, BET surface area significantly influenced the basicity of catalysts and larger BET surface area was favorable for increasing the exposed basic sites. The total basicity of various MgO catalysts was in the order of MgO-P-Na_2_CO_3_-3.14 ˃ MgO-P-Na_2_CO_3_-3 ˃ MgO-P-NaOH + Na_2_CO_3_-1/3 ˃ MgO-P-NaOH + Na_2_CO_3_-3/1 ˃ MgO-P-NaOH-3 ˃ MgO-P-NaOH-6, which was in good accordance with the variation tendency of BET surface area.

**Table 3:** The basicity distribution of different MgO catalysts.

Catalysts	Number of basic sites (mmol/g)		Total basicity (mmol/g)
	Weakly basic sites	Moderately basic sites	
MgO-P-NaOH-3	1.403	0.656	2.059
MgO-P-NaOH-6	0.985	0.549	1.534
MgO-P-NaOH + Na_2_CO_3_-3/1	1.442	0.825	2.267
MgO-P-NaOH + Na_2_CO_3_-1/3	1.521	0.915	2.436
MgO-P-Na_2_CO_3_-3	1.524	1.123	2.647
MgO-P-Na_2_CO_3_-3.14	1.315	1.544	2.859

According to the CO_2_-TPD analysis, the correlation between the amount of moderately basic sites and the DMC yield is exhibited in Fig. 8B in order to disclose the relationship of structures and performance. It was clear that the DMC yield increased linearly with the enhanced moderately basic sites amount, indicating that the moderately basic sites of MgO catalysts played the crucial role in DMC synthesis. We already realized that the intermediates (HEMC) could be produced without the presence of MgO and it could be deduced that the moderately basic sites were the active center for HEMC transesterification with methanol. Accordingly, the DMC yield was closely related to the amount of moderately basic sites. The MgO-P-Na_2_CO_3_-3.14 had the largest amount of moderately basic sites, leading to the highest DMC yield among all the MgO catalysts.

#### Surface chemical states and compositions of catalysts

**Figure 9:**
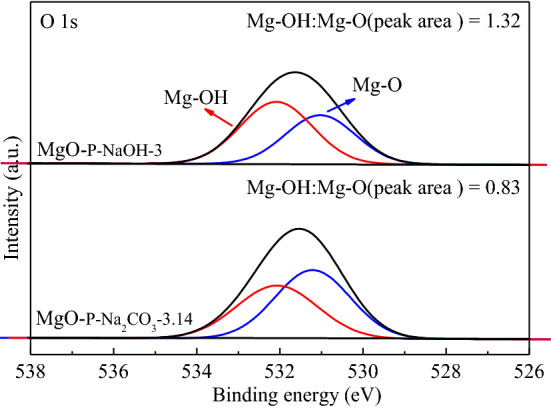
O 1 s XPS spectra of MgO_-P-NaOH-3_ and MgO_-_P-Na_2_CO_3_-3.14 catalysts.

The surface state of oxygen over MgO catalysts is evaluated by XPS and the O 1 s XPS spectra of MgO-P-NaOH-3 as well as MgO-P-Na_2_CO_3_-3.14 catalysts are displayed in Fig. 9. The O 1 s spectra could be divided into two peaks. The first peak at about 532.1 eV was derived from the chemisorbed –OH groups on the surface of MgO, while the second peak at around 531.0 eV was associated with lattice oxygen arose from MgO^54,55^. The peak area ratio of Mg-OH to Mg-O was calculated and exhibited in Fig. 9. It was clearly seen that the peak area ratio of Mg–OH to Mg–O was 1.32 for MgO-P-NaOH-3, which was significantly higher than that of MgO-P-Na_2_CO_3_-3.14 (0.83), indicating that MgO-P-NaOH-3 had larger number of –OH groups, while MgO-P-Na_2_CO_3_-3.14 sample had more Mg–O pairs. As discussed in CO_2_-TPD section, the amount of weakly basic sites was closely related to –OH groups adsorbed on the catalyst surface and the amount of moderately basic sites was associated with Mg–O pairs. Therefore, it was concluded that MgO-P-NaOH-3 had larger amount of weakly basic sites, while MgO-P-Na_2_CO_3_-3.14 had greater amount of moderately basic sites. This finding was in good agreement with the previous CO_2_-TPD analysis and the reaction results. Fig. S1 exhibits Mg 1 s spectra of MgO-P-NaOH-3 and MgO-P-Na_2_CO_3_-3.14 catalysts. Both of them showed Mg 1 s peaks centering at 1304.0 eV, which was in good agreement with the previous XRD patterns (Fig. 3B) displaying the presence of cubic MgO phase on the calcined catalysts.

#### Micro morphology and structure analysis of catalysts

**Figure 10:**
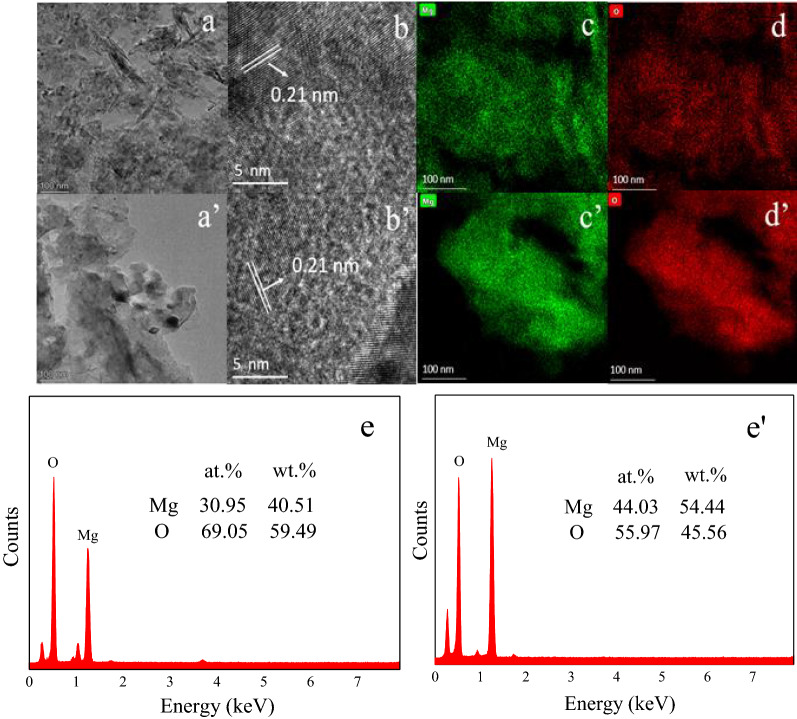
TEM images of MgO-P-NaOH-3 (**a**) and MgO-P- Na_2_CO_3_-3.14 (**a’**). HR-TEM images of MgO-P-NaOH-3 (**b**) and MgO-P- Na_2_CO_3_-3.14 (**b’**). TEM mapping analysis of Mg and O elements on the surface of MgO-P-NaOH-3 (**c**,**d**) and MgO-P- Na_2_CO_3_-3.14 (**c’**–**d’**). EDS analysis of MgO-P-NaOH-3 (**e**) and MgO-P- Na_2_CO_3_-3.14 (**e’**).

TEM images of MgO-P-NaOH-3 and MgO-P-Na_2_CO_3_-3.14 are compared in Fig. 10a and (a'). The MgO-P-NaOH-3 catalyst contained both plate-like and rod-like particles, while the MgO-P-Na_2_CO_3_-3.14 sample mainly included flaky-like particles. HR-TEM images of MgO-P-NaOH-3 (Fig. 10b) and MgO-P-Na_2_CO_3_-3.14 (Fig. 10b') indicated a lattice spacing of 0.21 nm, which was derived from the (200) plane of MgO. The TEM mapping results obviously demonstrated that Mg (Fig. 10c,c’) and O (Fig. 10d,d’) elements distributed homogenously on these two samples. The EDS analysis presented that 30.95 at.% Mg and 69.05 at.% O existed on the surface of MgO-P-NaOH-3 (Fig. 10e), while 44.03 at.% Mg and 55.97 at.% O stayed on the surface of MgO-P-Na_2_CO_3_-3.14 (Fig. 10e’). It could be calculated that the atomic ratio of O to Mg was much higher than 1/1 for these two samples, especially for MgO-P-NaOH-3 prepared with 3.0 mol/L NaOH aqueous solution. This phenomenon could be explained by chemisorbed -OH groups on MgO surface, which was supported by XPS analysis. Similar results were also reported by Ashok et al.^56^. Greater ratio of O to Mg was on behalf of higher –OH groups adsorbed on the surface of MgO, leading to more weakly basic sites and poorer activity.

### The proposed reaction mechanism of DMC synthesis via the transesterification of EC with methanol

**Figure 11:**
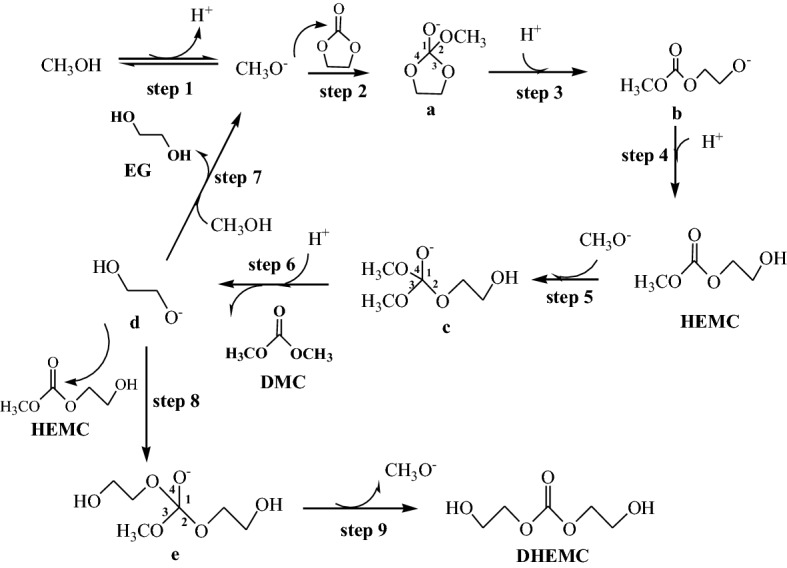
The proposed reaction mechanism of DMC synthesis via the transesterification of EC and methanol.

The proposed reaction mechanism of DMC synthesis via the transesterification is displayed in Fig. 11. Methanol was first deprotonated to form CH_3_O^−^ ion (**step 1**). Afterwards, CH_3_O^−^ ion served as a nucleophile to attack the carbonyl group in EC, leading to the generation of intermediate **a** with four C–O bands (**step 2**), which was thermodynamically unstable. It is necessary to break one of C–O bands to obtain a steady state. The marked 3 and 4 C–O bands were more easily to be broken compared with other two C–O bands. Due to the symmetry of intermediate **a,** only one intermediate **b** arose from the cleavage of 3 or 4 C–O band was produced (**step 3**). Then, the intermediate **b** combined with H^+^ in the reaction system to generate HEMC intermediate (**step 4**). Thereafter, the intermediate **c** was formed via the nucleophile addition reaction of HEMC intermediate with CH_3_O^−^ ion (**step 5**). Subsequently, the final product DMC and the intermediate **d** were generated through the cleavage of the marked 2 C-O band in intermediate **c** (**step 6**), followed by the combination of the intermediate **d** with H^+^ produced ethylene glycol (EG) (**step 7**). In addition, the carbonyl group in HEMC intermediate may also be attacked by the intermediate **d**, resulting in the formation of intermediate **e** (**step 8**). Finally, the removal of CH_3_O^−^ ion from intermediate **e** led to the generation of DHEMC product (**step 9**). The GC–MS was used to determine the intermediate products and the GC–MS graph is exhibited in Fig. S2. There existed six products in liquid phase, including the excessive methanol, the DMC and EG products, the HEMC and DHEMC intermediates as well as unreacted EC. Therefore, the GC–MS analysis confirmed the presence of HEMC and DHEMC intermediates in liquid products, indicating that the proposed reaction mechanism of DMC synthesis via the transesterification was reasonable.

### Catalyst reusability and stability

**Figure 12:**
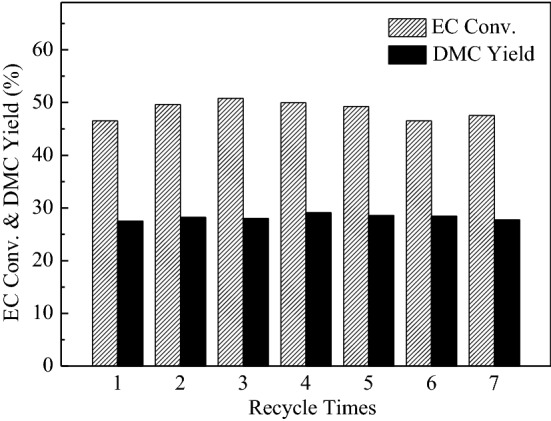
The effect of recycle times on EC conversion and DMC yield. Reaction conditions: EC/MeOH = 1/6, catalyst amount = 3 wt%, reaction temperature = 68 °C, reaction time = 0.5 h.

Based on the above results and discussion, MgO-P-Na_2_CO_3_-3.14 exhibited the best catalytic activity among all the catalysts. In this section, catalyst stability of MgO-P-Na_2_CO_3_-3.14 sample was studied. The effect of recycle times on EC conversion and DMC yield is displayed in Fig. 12. It was obvious that the MgO-P-Na_2_CO_3_-3.14 catalyst could be reused for seven times. The EC conversion was maintained at about 47% with 27% yield of DMC. Therefore, the MgO-P-Na_2_CO_3_-3.14 catalyst exhibited not only superior activity, but also outstanding reusability, and thus showcasing the excellent prospects in DMC synthesis. The CO_2_-TPD curves of MgO_-P-Na2CO3-3.14_ catalyst before and after reaction are compared in Fig. S3. It was obvious that the peak area of both weakly and moderately basic sites maintained almost constant before and after reaction, indicating that the basic sites amount kept nearly unchanged.

The MgO-P-Na_2_CO_3_-3.14 catalyst was also tested in a continuous fixed-bed reactor and the results are exhibited in Fig. S4. It was obvious that the EC conversion kept at 36.3% with DMC yield of 10.7% even after 100 h reaction, indicating that MgO-P-Na_2_CO_3_-3.14 displayed excellent stability for transesterification of EC with methanol.

## Conclusion

A series of MgO catalysts obtained by different preparation methods, such as hydration synthesis, thermal decomposition, combustion, sol–gel and co-precipitation, were prepared for DMC synthesis. Among them, MgO-P-Na_2_CO_3_-3.14 from excess Na_2_CO_3_ precipitation displayed superior catalytic activity and was reused for seven times without obvious deactivation. The DMC yield, which was closely related to the amount of moderately basic sites, was as high as 69.97% at 68 °C. We recognized that the transesterification of EC with methanol could be divided into two steps, including the ring-opening reaction of EC and the transesterification of HEMC with methanol. MgO came from NaOH precipitant had better ring-opening capability, while MgO catalyst received by Na_2_CO_3_ precipitant had superior transesterification ability. The TG–DTA results disclosed that the as-prepared catalyst arose from dried precursors with more crystalline magnesium carbonate was advantageous for enhancing DMC yield. The XPS and EDS analysis indicated that MgO-P-Na_2_CO_3_-3.14 had rich Mg-O pairs on the surface with Mg/O ratio about 1, resulting in more moderately basic sites. Mg-O pairs were the active center for HEMC transesterification to produce DMC. In a word, as-synthesized MgO-P-Na_2_CO_3_-3.14 with larger crystallite sizes, greater BET surface area and mesopore volume, larger moderately basic sites amount showcased excellent activity and bright prospect in industrial application.

## Supplementary Information

Supplementary Information.
