# Supplementary material for: Excess soluble alkalis to prepare highly efficient MgO with relative low surface oxygen content applied in DMC synthesis
Source: Sci Rep. 2021 Oct 22;11:20931. doi: 10.1038/s41598-021-00323-5 (PMC8536740; doi:10.1038/s41598-021-00323-5)
Supplement: Supplementary file 1 — Supplementary Information. [file 41598_2021_323_MOESM1_ESM.docx]

**Supporting Information**

**Excess soluble alkalis to prepare highly efficient MgO with relative low surface oxygen content applied in DMC synthesis**

**Ju Liu^ac#^, Fei Chen^ac#^, Wenbing Yang^b^, Jianjun Guo^b^,** **Guangwen Xu^ac^, Fenglei Jia^b*^, Lei Shi^ac*^**

*^a^ Key Laboratory on Resources Chemicals and Materials of Ministry of Education, Shenyang University of Chemical Technology, Shenyang 110142, China.*

*^b^* *Shandong Shida Shenghua Chemical Group, Dongying 257000, China.*

*^c^* *Institute of Industrial Chemistry and Energy Technology, Shenyang University of Chemical Technology, Shenyang 110142, China.*

^*^ Corresponding author, Tel/Fax: +(86)-024-89388216;

^#^ These authors are equal to this work.

E-mail address: fenglei@sinodmc.com (Fenglei Jia)

shilei@syuct.edu.cn (Lei Shi)

**Fig. S1.** Mg 1s spectra of MgO_-P-NaOH-3_ and MgO_-P-Na2CO3-3.14_ catalysts.

**Fig. S2.** GC-MS graph of liquid products.

**Fig. S3.** CO_2_-TPD curves of MgO_-P-Na2CO3-3.14_ catalyst before and after reaction.

**Fig. S4.** The continuous fixed-bed reaction results over MgO_-P-Na2CO3-3.14_ catalyst. Reaction conditions: catalyst weight = 1.0 g, reaction temperature = 75-77 ℃, atmospheric pressure, MeOH/EC molar ratio = 6/1, weight hourly space velocity (WHSV) = 5.0 h^-1^.
